# Trauma of the Frontal Region Is Influenced by the Volume of Frontal Sinuses. A Finite Element Study

**DOI:** 10.3389/fphys.2017.00493

**Published:** 2017-07-11

**Authors:** Srbislav S. Pajic, Svetlana Antic, Arso M. Vukicevic, Nenad Djordjevic, Gordana Jovicic, Zivorad Savic, Igor Saveljic, Aleksa Janović, Zoran Pesic, Marija Djuric, Nenad Filipovic

**Affiliations:** ^1^Clinic for Neurosurgery, Clinical Center of Serbia Belgrade, Serbia; ^2^Center for Radiological Diagnostics, School of Dentistry, University of Belgrade Belgrade, Serbia; ^3^Laboratory for Anthropology, Institute of Anatomy, School of Medicine, University of Belgrade Belgrade, Serbia; ^4^Faculty of Engineering, University of Kragujevac Kragujevac, Serbia; ^5^Research and Development Center for Bioengineering, Kragujevac Kragujevac, Serbia; ^6^Faculty of Information Technology, Belgrade Metropolitan University Belgrade, Serbia; ^7^Structural Integrity Theme, Institute of Materials and Manufacturing, Brunel University London, United Kingdom; ^8^Emergency Radiology Department, Clinical Center of Serbia-Emergency Hospital Belgrade, Serbia; ^9^Department for Maxillofacial Surgery, School of Medicine, University of Nis Nis, Serbia

**Keywords:** frontal sinus, fracture, frontal bone, finite element analysis, modeling

## Abstract

Anatomy of frontal sinuses varies individually, from differences in volume and shape to a rare case when the sinuses are absent. However, there are scarce data related to influence of these variations on impact generated fracture pattern. Therefore, the aim of this study was to analyse the influence of frontal sinus volume on the stress distribution and fracture pattern in the frontal region. The study included four representative Finite Element models of the skull. Reference model was built on the basis of computed tomography scans of a human head with normally developed frontal sinuses. By modifying the reference model, three additional models were generated: a model without sinuses, with hypoplasic, and with hyperplasic sinuses. A 7.7 kN force was applied perpendicularly to the forehead of each model, in order to simulate a frontal impact. The results demonstrated that the distribution of impact stress in frontal region depends on the frontal sinus volume. The anterior sinus wall showed the highest fragility in case with hyperplasic sinuses, whereas posterior wall/inner plate showed more fragility in cases with hypoplasic and undeveloped sinuses. Well-developed frontal sinuses might, through absorption of the impact energy by anterior wall, protect the posterior wall and intracranial contents.

## Introduction

It is well known that anatomy of the frontal sinuses varies individually, in terms of different volume and shape. About 4% of the population does not have frontal sinuses and other 4 to 5% have only small rudimentary air cells (Aydinlioǧlu et al., 2003; Pondé et al., 2003; Kalavrezos, 2004; Montovani et al., 2006). Normally developed frontal sinuses are usually about 28–30 mm in height, 24–28 mm in width and 20 mm in depth, creating a space of 5–7 ml (Amine and Anand, 2015). A small/hypoplasic frontal sinus is an underdeveloped sinus cavity, size of a smaller peace, located above the frontal maxillar processus. Enlarged frontal sinuses could be classified into three categories which include occurrence of symptoms as well: (1) hyper-(pneumatised)-sinus - developed beyond the established limits of normal frontal sinus aeration in an asymptomatic patient, (2) pneumosis dilatans–the entire sinus or a segment develops beyond the confines of the frontal bone and encroaches upon the adjacent structures, and (3) pneumocele—produce regional signs and symptoms relative to sinus overgrowth and causes thinning of the overlying bone (Urken et al., 1987).

Frontal sinus is directly connected with the anterior cerebral fossa, nose and orbital roof, giving a great complexity to the trauma of this region. Generally, fractures of the frontal bone are associated with high impact trauma and dynamic forces, such as traffic accidents, assaults, and sport accidents (May, 1970; Tiwari et al., 2005; Bell et al., 2007; Mithani et al., 2009). The injuries of the frontal region are varying, as they range from isolated fractures of the anterior sinus' wall, to very complex fractures involving the orbit and skull base (Strong et al., 2006; Holier et al., 2010; Dimitrijevic et al., 2014). Fracture pattern and its complexity depend on many factors, such as impact force intensity and direction, site of impact, and frontal bone quality.

However, the influence of the frontal sinus volume on the stress distribution and fracture pattern at impact within the frontal region is still unclear. Some experimental studies on cadavers and artificial head models investigated influence of impact on facial bones, and its biomechanical response (Nyquist et al., 1986; Allsop et al., 1988; Cormier and Manoogian, 2010). But the studies were limited to small number of cadavers and didn't consider the influence of variations in human anatomy and impact force, since the experiments are not repeatable and commonly ended with the specimen fracture. These limitations motivated researchers to apply the computers and numerical procedures such as Finite Element Analysis (hereinafter *FEA*) for studying head injury. Using realistic geometry obtained from medical scans and experimentally determined material properties of the tissues, *FEA* allows for calculation of head physical response (displacements, stresses, strains etc.) under an arbitrary conditions (loads, constraints), which are very difficult (if at all) to estimate in the experimental conditions. A number of human-head models were developed, validated and proposed for studying the human head and brain injuries (Zhang et al., 2001; Samaka and Tarlochan, 2013; Asgharpour et al., 2014). However, most of these studies were focused on brain injuries assuming simplified human head models that did not included the frontal sinuses. Song et al. (2015) were the first who have studied the dynamic response of the skull under blunt frontal, zygomatic and maxillary impacts, considering the cases with and without sinuses. The results of this study demonstrated that, in forehead impact, the frontal sinuses significantly affected the distribution of stress and strain in the skull. However, this study did not consider the influence of various sinus volumes on the stress distribution in the frontal bone and cranial base.

Therefore, the aim of the study was to evaluate the impact of the frontal sinus volume on the stress distribution and fracture pattern in the frontal region, by means of *FEA*.

## Materials and methods

### Test cases considered and methodology for development of numerical models

The patient specific anatomical models used in this study were developed from the *CT* scans of the human head, with normally developed frontal sinuses. The scans were selected from the database of the Emergency Hospital/Emergency Radiology Department of Diagnostics-Clinical Center of Serbia *CT* scanner (Aquilion™ PRIME 160 slices AIDR 3D integrated, Toshiba Medical System Tehnologies, Nasu, Japan, 72kW, 13,6mGy). This study was carried out in accordance with the recommendations of Helsinki declaration and the guidelines of the International Committee of Medical Journal Editors, with approvals from Ethic Committee of the Clinical Centre Serbia (Date 24. 11. 2016. No. 195/43) and Ethics Committee of the Faculty of Medicine University of Nis (Date 27 12 2016 No. 12-14532-2/6). Moreover, all subjects gave written informed consent in accordance with the Declaration of Helsinki.

**Figure 1:**
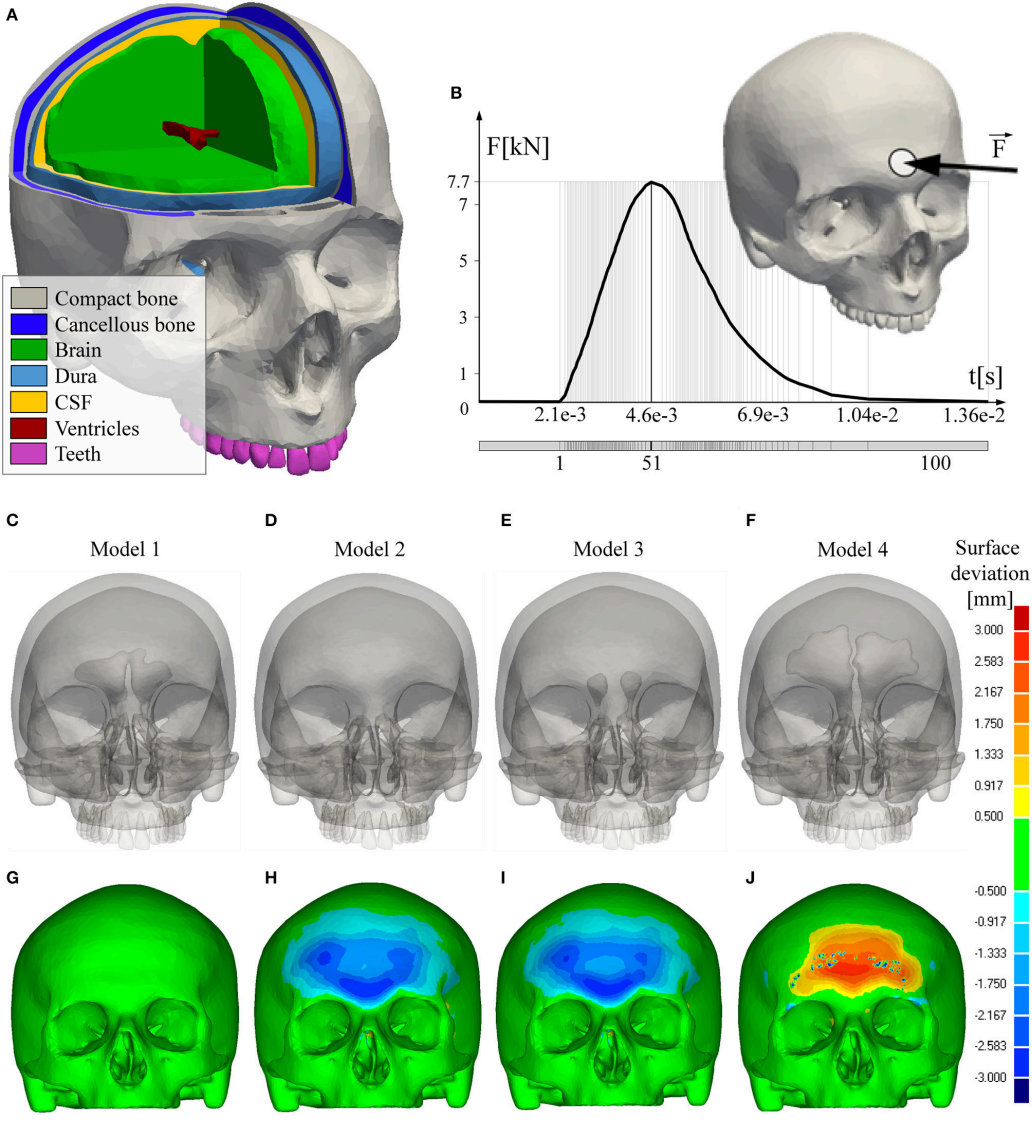
FEA models and boundary conditions. **(A)** Considered materials-tissues; **(B)** Loading conditions; **(C)** Reference model with normally developed frontal sinus cavities; **(D)** Model without frontal sinuses; **(E)** Model with hypoplasic frontal sinus cavities; **(F)** Model with hyperplasic frontal sinus cavities; **(G–J)** Deviation of the FEA models from the reference model.

Image reconstruction was performed by using a filtered back-projection type cone beam reconstruction algorithm (ConeXact, Toshiba Medical Systems, Nasu, Japan). The considered patient was a male adult (27 year old) with no sinus pathology. The 160-slice *CT* scans were acquired with the 0.5 mm isotropic resolution of 350 × 350 pixels with the pixel size 0.5 × 0.5 mm^2^ and 0.5 mm slice thickness. Three dimensional reconstruction of human head model from its *CT* scans (Figure 1A) was performed by using the Mimics software (Materialize, Leuven, Belgium), version 10. The first step was obtaining the mask of cortical bone, than the masks of diploe and teeth. Subsequently, the masks of the following intracranial tissues were created: dura mater, cerebro-spinal fluid (*CSF*), brain tissue and ventricles. All developed masks were converted from the volume into the stereolithography (*STL*) format by using the Mimics *STL*+ module. The quality of *STL* mesh was improved by reducing the number and fixing quality of the triangles by using the REMESH module. The described model represents Model 1- with normally developed frontal sinuses and it was used as a reference model for the further analyses (Figure 1C).

Using the *CT* scans and *STL* meshes of the model with normally developed sinuses, three additional virtual models were generated. Model 2 represents the human skull without frontal sinuses (Figure 1D), Model 3 is with small- hypoplasic (Figure 1E), and Model 4-with large frontal sinuses (Figure 1F). Anatomical skull geometries were achieved by corresponding contraction and expansion of the Model 1 sinus cavity, i.e., by adding or deleting the pixels of cortical and medullary bone. The modifications were made with respect to the anatomies of patients, who had frontal bone with hypoplasic, enlarged, and with undeveloped sinuses, using the database available in the Clinical Center Serbia. The categorisation criteria for defining the hypoplasic, normal and enlarged sinus cavities were based on the sinus volume determined by *CT* and data available in the open literature (Yüksel Aslier et al., 2016). Namely, the hypoplasic frontal sinus is defined as a sinus with volume size lower than 1131.25 mm^3^. Volume of the normally developed frontal sinus is between 1131.25 mm^3^ and 3328.50 mm^3^, whilst the volume of the enlarged or hyperplasic frontal sinus is over 3328.50 mm^3^. Hyperplasic frontal sinus selected for this study was classified as hyper-(pneumatised)-sinus- one that is developed beyond the established limits of normal frontal sinus aeration in an asymptomatic patient (Urken et al., 1987).

The resulting *STL* files of the four models developed were imported into the CATIA V5 software R20 (Dassault Systèmes, Velizy-Villacoublay, France; modules Digitized Shape Editor and Quick Surface Reconstruction modules) in order to obtain their NURBS surfaces representation which is suitable for the further structural analysis. The obtained solid models were finally imported into ANSYS software (SASI, Canonsburg, PA, United States of America), version 14.5.7, for generating *FEA* mesh and conducting structural analysis.

### *FEA* models and material properties

Material characteristics of the bone, teeth and intracranial contents were taken from the literature (Mao et al., 2013; Asgharpour et al., 2014; Antic et al., 2015; Song et al., 2015) and are shown in Table 1. The cortical bone, diploe, dura, ventricles and teeth were modeled as linear elastic material, described with Young's modulus (E), Poisson's ratio (ν) and density. Brain and cerebro-spinal fluid (*CSF*) were modeled using the viscoelastic material model described with the following equation: G(t) = G_∞_+(G_0_+G_∞_)·e^βt^, where G_∞_ is long-time shear modulus, G_0_ is short-time modulus, β is decay coefficient and t is time. In order to estimate a risk from skull fracture, it was necessary to adopt the limiting values of compressive and tensile stresses of cortical bone. In the present study, the adopted values of compressive and tensile ultimate strength were σ_cs_ = 133 MPa and σ_ts_ = 92 MPa, respectively (Antic et al., 2015). The four *FEA* models were discretized into the fine volume mesh, using the linear tetrahedron elements (Tet4) available in ANSYS Meshing module. The *FEA* models of the Model 1, Model 2, Model 3 and Model 4 were defined with 665614, 663517, 664729, and 666815 tetrahedron elements, respectively. For the considered models, values of the considered mesh quality indicatros were: Jacibian = 1; average Element quality (the ratio of the volume to the square root of the cube of the sum of the square of the edge lengths) was 0.83 with the standard deviation of 0.142; average Aspect ratio was 2.136 with the standard deviation of 0.70. These values were achieved iteratively, by refining the mesh until coarsening of the mesh does not disturb the stress field (particularly, the changes of the stress values between the last two successive refinements were ~2%).

**Table 1:** Mechanical properties of the considered materials.

**Material**	**Property**	**E (Mpa)**	**ν**	**ρ (kg/m^3^)**	***K* (MPa)**	***G*_0_ (kPa)**	***G*_∞_ (kPa)**	***B* (kPa)**
Cortical bone	Elastic	15,000	0.21	1,900	–	–	–	–
Diploe	Elastic	4,600	0.05	1,500	–	–	–	–
Brain	Viscoelastic	–	–	1,040	2,190	6	0.1	80
Dura	Elastic	31.5	0.35	1,100	–	–	–	–
CSF	Viscoelastic	–	–	1,040	2,190	0.5	0.1	80
Ventricles	Elastic	31.5	0.315	1,100	–	–	–	–
Teeth	Elastic	18.60	0.31	2,100	–	–	–	–

*E, Young's modulus; ν, Poisson's ratio; ρ, Density; K, Bulk modulus; G_0_, Short-time shear modulus; G_∞_, Long-time shear modulus*.

### Boundary conditions and structural strength analysis

All of the simulations were conducted in ANSYS as the transient structural analysis within 100 time-steps (Figure 1B). A blunt trauma was applied as an impact force acting perpendicularly to the forehead over a circular area 2 cm in diameter (Figure 1B). The impact force intensity of 7.7 kN was adopted from the experimental study conducted by Nahum (Nahum et al., 1977) and used in previous *FEA* studies of the human head impact (Zong et al., 2006; Mao et al., 2013). The nodes of the external occipital protuberance of the investigated skull were fixed in all degrees of freedom. The distribution of the effective-von Mises stress (a measure of the intensity of the multiaxial stress state, Pruitt and Chakravartula, 2011), Maximum and Minimum stress, estimated at the peak moment of the impact force, were analyzed. The fracture risk for the developed models was calculated by using the Maximum Principal Stress Criterion- *MPSC* (Gross and Seelig, 2011; Antic et al., 2015; Zelic et al., 2015). Following the *MPSC*, it was assumed that the skull failure occurs when a positive (compressive) component of the principal stress exceeds the tensile strength σ_ts_ or when a negative component of the principal stress is less than the compressive strength σ_cs_. Therefore, the failure index (*FI*) was calculated using the principal stresses σ_1_ (maximum) and σ_3_ (minimum) as: *FI*(σ_i_) = σ_i_/σ_ts_ if σ_i_ > 0 and *FI*(σ_i_) = σ_i_/σ_cs_ otherwise.

## Results

**Figure 2:**
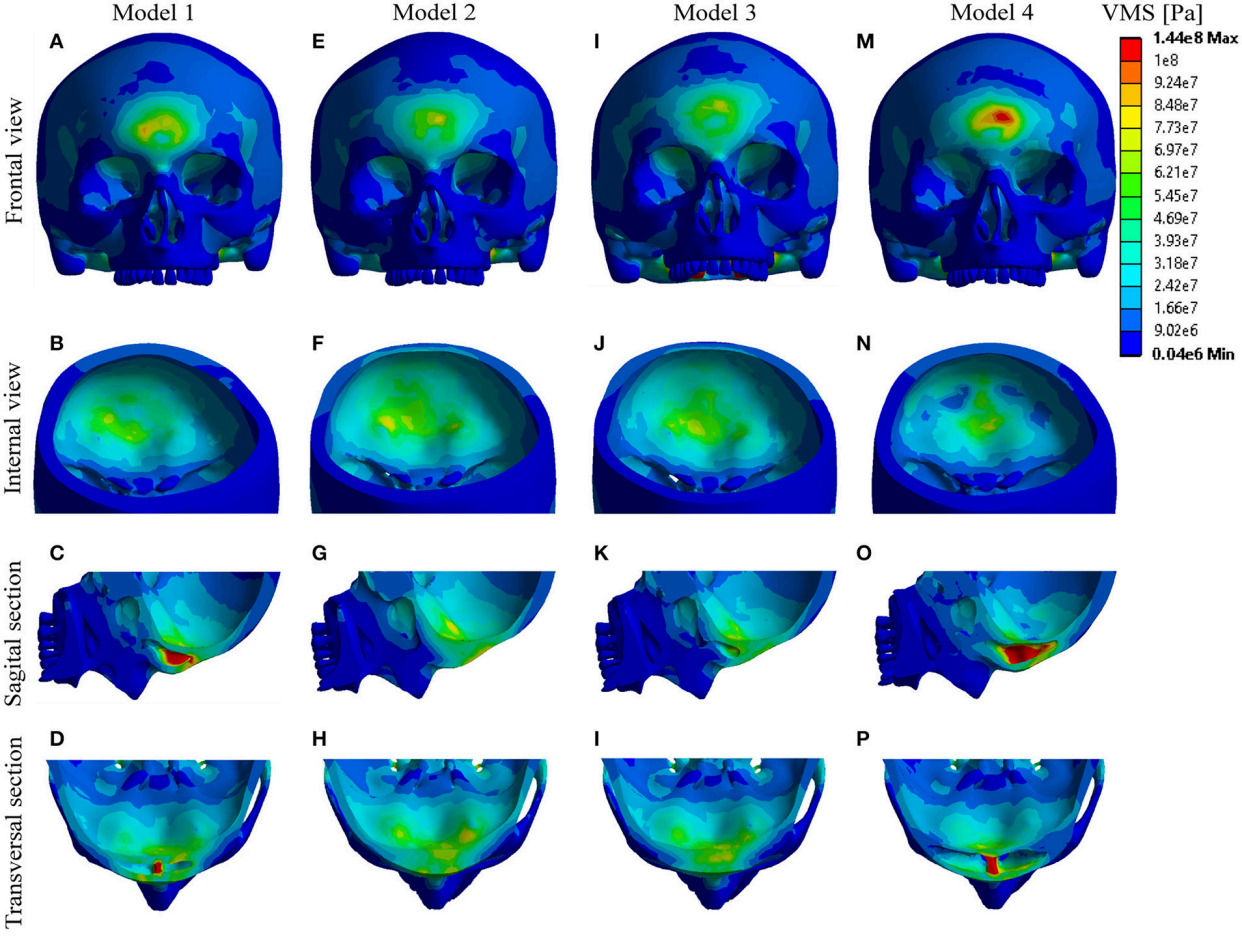
Distribution of effective-Von Mises stress. **(A–D)** Model 1; **(E–H)** Model 2; **(I–L)** Model 3; **(M–P)** Model 4.

The results demonstrate the distribution of the impact-induced stress in the frontal region, depending on the volume of the sinus cavities. Distributions of the effective (von Mises) and principal stresses (compressive and tensile stress) with calculated failure indices are estimated at the time distance that corresponds to the peak of the impact force, and presented chromatically on the figures below (Figures 2, **4**, **5**).

Chromatic analysis of the distributed effective-von Mises (*VMS*) is presented in the Figure 2. The results obtained show that, following the impact, the extreme values of the *VMS* occurred in vicinity of impact (including anterior sinus wall) in all the models. The stress concentration also occurred in the regions of septum, posterior sinus wall and frontal part of the cranial base. Considering the anterior sinus wall, the highest level of stress was reached in Model 4, whilst the maximum values of the obtained stress appeared in the models in the following decreasing order: Model 4 > Model 1 > Model 3 > Model 2. In contrast, considering the posterior sinus wall/interior cortical plate, and frontal part of the cranial base, the order of the Models with decreasing stress values was totally opposite: Model 2 > Model 3 > Model 1 > Model 4 (Figure 2).

**Figure 3:**
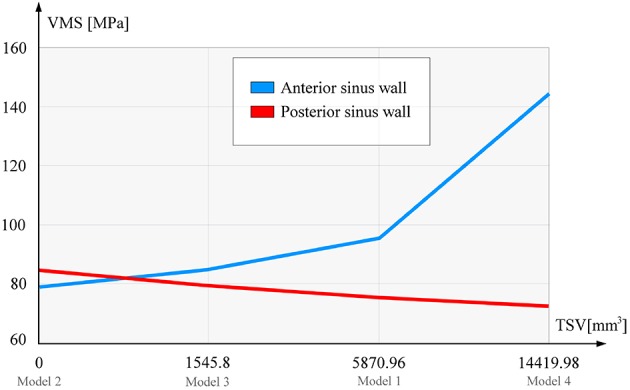
Sinus volume and maximum Von Mises stress ratio. TSV, total sinuse volume (left+right sinus cavity); VMS, Von Mises stress (presented are the maximum values).

Figure 3 shows the ratio of the frontal sinus volume and maximum values of the concentrated *VMS*. It is demonstrated that the increase in the volume of the sinuses was followed with the increase of the *VMS* at the anterior sinus wall, which was more significant than the decrease of the *VMS* at the posterior sinus wall. However, regardless the small differences in the maximum *VMS* measured at the posterior sinus wall/ depending on the sinus volume, the differences in the distribution of the *VMS* among the models were obvious (Figures 2B–N).

**Figure 4:**
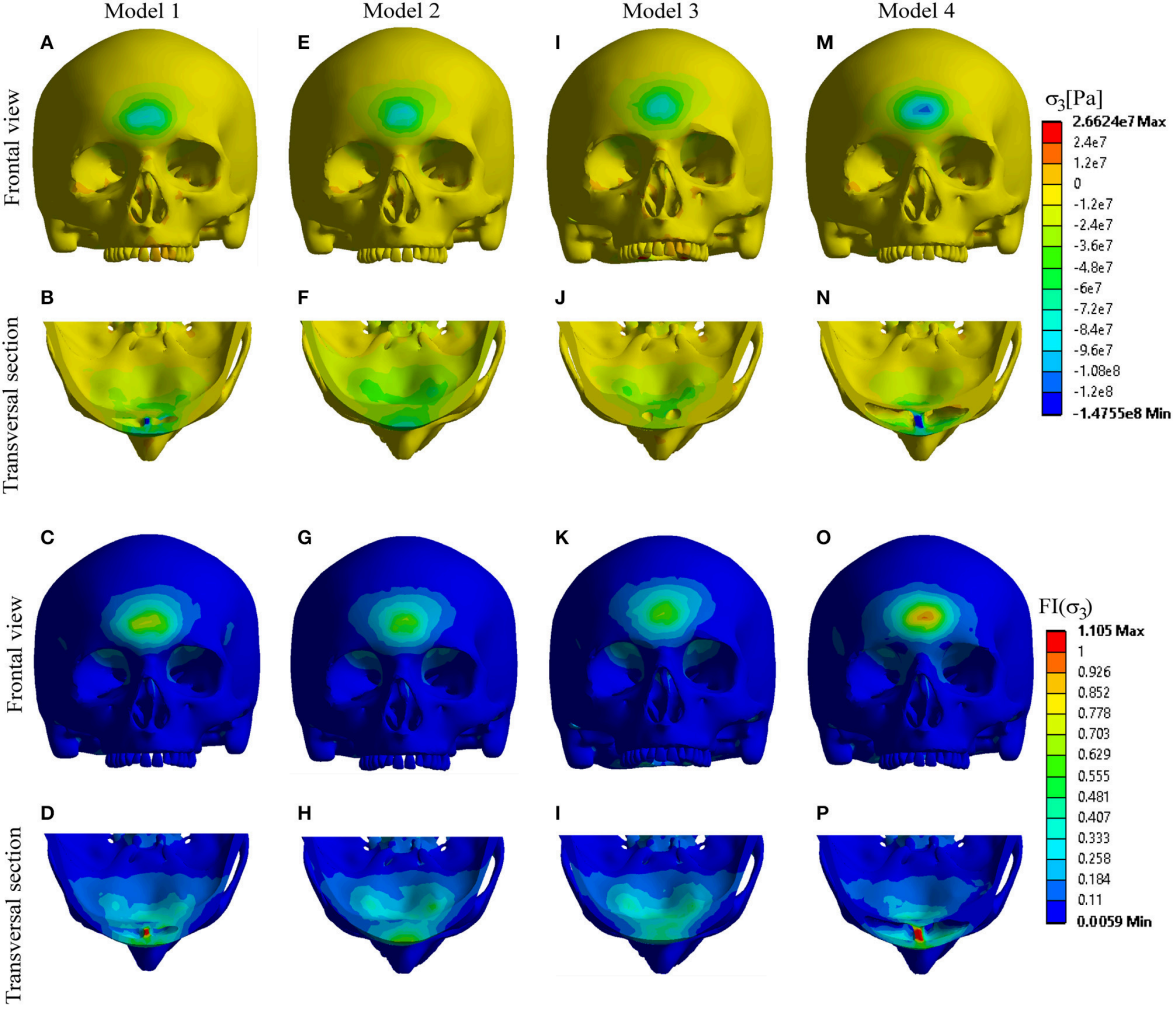
Distribution of compressive stress and *FI*-compression. **(A–D)** Model 1; **(E–H)** Model 2; **(I–L)** Model 3; **(M–P)** Model 4.

**Figure 5:**
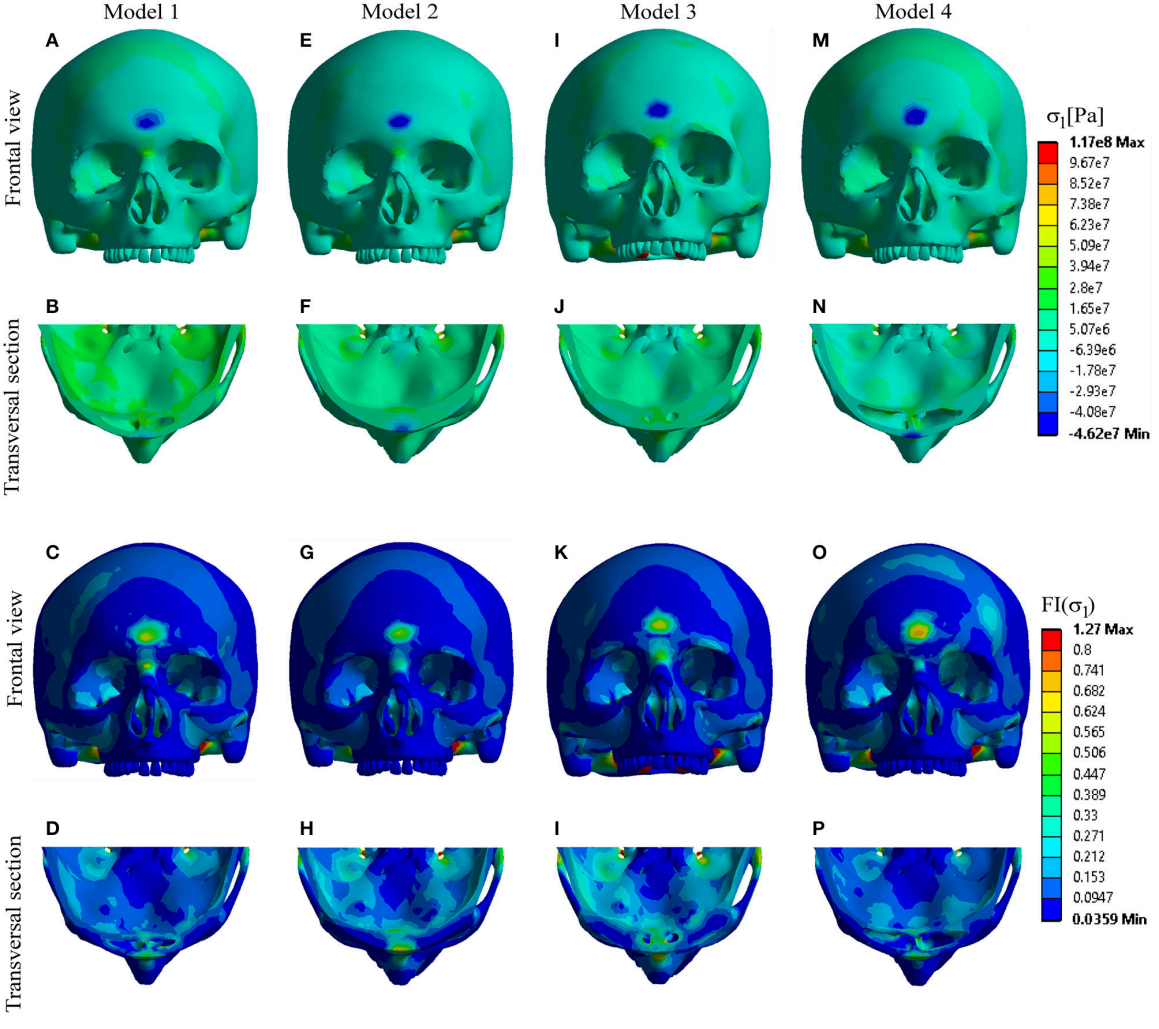
Distribution of tensile stress and *FI*-tension. **(A–D)** Model 1; **(E–H)** Model 2; **(I–L)** Model 3; **(M–P)** Model 4.

Analysis of the principal stresses with calculation of their failure indices (*FI*) showed the following:

- Compressive stress mostly concentrated at the point of impact, and in the septal regions of the Model 1 and Model 4. Based on the obtained results and the calculated values of the *FI*, fracture could be expected only in the frontal region of Model 4, and septal regions of the Models 1 and 4 (Figure 4).
- Tensile stress distribution was observed mostly at the posterior sinus wall and frontal part of the cranial base, reaching the maximum values in Model 2 and Model 3, respectively. However, none of the Models showed a failure, which suggests that the failure could be expected only at higher levels of the impact force (Figure 5).

## Discussion

Numerous assumptions have been proposed in order to explain the function of the paranasal sinuses and many of them were discarded (Blaney, 1990; Rhys-Evans, 1992; Rae and Koppe, 2004; Keir, 2009). Recently, Kellman and Schmidt (2009) have proposed a theory that paranasal sinuses serve as a crumple zones with a role to provide a compressible or fragmentable barrier that absorbs and disperses energy through the destruction and/or deformation of the crumple zone itself. In an experimental study with nine cadavers, they showed that when trauma was directed to the globe, the thin orbital floor fractured referentially into the maxillary sinus, thereby protecting the globe from rupture. When the ethmoid and maxillary sinuses were eliminated, similar trauma caused ruptures of the globes. Furthermore, they assumed that there is a similar way in which frontal sinuses protect the frontal lobe.

Inspired with the theory of paranasal sinuses as protective structures, Song et al. (2015) have validated two models of the human head: with and without paranasal sinuses, against three different impacts: impact to the forehead, zygoma and maxilla. In forehead impact, frontal sinuses significantly influenced the accumulation of stress in the nearby intracranial zone. Namely, stress in the nearby intracranial zone was higher in the model without sinuses by about 35% than in model with sinuses.

Yu et al. (2014) in an epidemiological study examined the interactions of the frontal sinuses with different volume and the brain in the setting of head trauma. They showed that the volume of the frontal sinuses was 33% less in patients with contusion than in patients without contusion of the brain, meaning that the frontal sinuses impart a protective advantage against frontal brain contusion.

In accordance with these data, the results of the present study showed that dynamics of head injury and stress distribution in the frontal region depend on the sinus volume, and confirmed the protective role of the frontal sinuses. After applying an impact force of 7.7 kN to the frontal region of the forehead, the stress occurred at the anterior sinus wall, region of sinus septum, at the posterior sinus wall and frontal region of cranial base in all the four Models. However, significant differences in the distribution of the extreme stress levels were found depending of the sinus volume. The anterior sinus wall showed the highest fragility in the case with hyperplasic sinus cavity due to high compressive stress. Based on the FI results (Figure 4), this stress could cause a fracture of the anterior wall of the hyperplasic sinuses and also the septa of the hyperplasic and normally developed sinuses. In contrast, posterior sinus wall/inner plate and frontal region of the cranial base demonstrated more fragility in cases with hypoplasic and even more in undeveloped sinuses, due to high tensile stress. However, the amounts of tensile stress in these regions were not sufficient to cause a failure (Figure 5). Fracture of the posterior wall or frontal cranial base require higher impact force, and will occur primarily in cases with hypoplasic or undeveloped sinuses.

In case of trauma of the frontal region with developed sinus cavity, anterior sinus wall and septa are predisposed to fracture due to the absorbed compressive stress, thereby minimizing the impact energy transmission to the frontal lobe. With hypoplasic or undeveloped sinus cavities, the anterior wall was less fragile, as the stress was conveyed to the posterior wall and frontal part of the cranial base. Although a higher impact force is required to cause a fracture of these regions, the distributed stress might also be transmitted onwards and affect the brain and vital structures. This is consistant with the results of the epidemiological study of Yu et al. (2014), where patients with brain contusion had 33% smaller sinuses than the patients without brain contusion.

Results of the study support the theory that frontal sinuses provide a compressible or fragmentable barrier that absorbs and disperses energy through the destruction and/or deformation of its anterior wall, thus protecting the posterior wall and sinus floor. Knowing that fractures of the posterior cortical plate and sinus floor usually cause injuries of the intracranial and orbital contents (Heller et al., 1989; Stanley, 1989), frontal sinuses do impart a protective role against the brain and eye trauma.

Understanding the dynamics of the skull in trauma is important for early recognition of severe cases, as well as for providing the future protection of the brain. In frontal trauma, brain contusion should be expected more likely in persons with smaller sinus cavities, therefore, special care should be taken in order to avoid possible oversights in diagnosis. The results of the study support the management of the frontal sinus fractures that follow the surgical approach of preservation and restoration of the posterior wall and sinus cavity, avoiding the radical procedures, whenever possible. This is of particular importance in people who are prone to regaining the impact in this region, such as individuals involved in various sports, or jobs with increased risk of being injured. Future investigations should be addressed to the development of adequate restorative materials and reconstructive strategies for preserving the integrity of the frontal sinus cavity.

In conclusion, our results showed that impact-induced stress distribution and fracture pattern in the frontal region highly depend on the volume of the frontal sinus cavities. Well-developed frontal sinuses might provide a survival benefit, by acting as “shock absorbers” that protect surrounding vital structures and intracranial contents. This study for the first time clarifies the mechanism of this, previously only presumed- protective role of the frontal sinuses.

## Author contributions

SP contributed to: original idea, literature search, study design, and writing a part of the text (in: Introduction/Discussion) SA contributed to: literature search, study design, and writing a part of the text (in: Introduction and Discussion); AV contributed to: data analysis, FE modeling and writing a part of the text (in: Materials and Methods and Results) ND contributed to: data analysis—FE analysis, data interpretation, and writing a part of the text (in: Materials and Methods Section) GJ contributed to: FE analysis, and writing a part of the text (in: Materials and Methods and Results) ZS contributed to: data collection, measuring the sinus volume, classification, and selection the patients which CT (in: Materials and Methods Section) IS contributed to: data analysis-FE analysis, data interpretation, and literature search, FE modeling; AJ contributed to: data collection, data interpretation, and literature search, FE modeling; ZP contributed to: literature search, data interpretation and critical revision of the manuscript; NF contributed to: study design, data interpretation and critical revision of the manuscript; MD contributed to: study design, writing a part of the text (in: Discussion) and critical revision of the manuscript.

### Conflict of interest statement

The authors declare that the research was conducted in the absence of any commercial or financial relationships that could be construed as a potential conflict of interest.
